# Effects of Late-Cenozoic Glaciation on Habitat Availability in Antarctic Benthic Shrimps (Crustacea: Decapoda: Caridea)

**DOI:** 10.1371/journal.pone.0046283

**Published:** 2012-09-27

**Authors:** Johannes Dambach, Sven Thatje, Dennis Rödder, Zeenatul Basher, Michael J. Raupach

**Affiliations:** 1 Zoologisches Forschungsmuseum Alexander Koenig, Bonn, Germany; 2 Ocean and Earth Science, National Oceanography Centre, University of Southampton, Southampton, United Kingdom; 3 Leigh Marine Laboratory, University of Auckland, Auckland, New Zealand; 4 Deutsches Zentrum für Marine Biodiversitätsforschung, Senckenberg am Meer, Wilhelmshaven, Germany; The Australian National University, Australia

## Abstract

Marine invertebrates inhabiting the high Antarctic continental shelves are challenged by disturbance of the seafloor by grounded ice, low but stable water temperatures and variable food availability in response to seasonal sea-ice cover. Though a high diversity of life has successfully adapted to such conditions, it is generally agreed that during the Last Glacial Maximum (LGM) the large-scale cover of the Southern Ocean by multi-annual sea ice and the advance of the continental ice sheets across the shelf faced life with conditions, exceeding those seen today by an order of magnitude. Conditions prevailing at the LGM may have therefore acted as a bottleneck event to both the ecology as well as genetic diversity of today's fauna. Here, we use for the first time specific Species Distribution Models (SDMs) for marine arthropods of the Southern Ocean to assess effects of habitat contraction during the LGM on the three most common benthic caridean shrimp species that exhibit a strong depth zonation on the Antarctic continental shelf. While the shallow-water species *Chorismus antarcticus* and *Notocrangon antarcticus* were limited to a drastically reduced habitat during the LGM, the deep-water shrimp *Nematocarcinus lanceopes* found refuge in the Southern Ocean deep sea. The modeling results are in accordance with genetic diversity patterns available for *C. antarcticus* and *N. lanceopes* and support the hypothesis that habitat contraction at the LGM resulted in a loss of genetic diversity in shallow water benthos.

## Introduction

With at least 350 different genera and more than 2,800 described species, caridean shrimps (Crustacea: Decapoda) represent a group of primarily marine crustaceans with a high degree of diversity in body form and occupied habitats [1]. Caridean shrimps are ecologically important in near shore habitats from tropical to high latitudes and have successfully colonized all marine habitats from shallow waters to abyssal plains and hydrothermal vents [1], [2]. In addition to the marine species, about 650 species have also successfully invaded brackish and freshwater habitats, particularly highly diverse in tropical and subtropical areas [1], [3].

Interestingly, only about a dozen caridean shrimp species are known from the Southern Ocean [4]–[7], with only three shrimp species left on the high-Antarctic continental shelves, where temperatures are below zero all year round (for review see [8]). Although they are low in species number, in terms of abundance these three shrimp species represent a major component of the mobile benthic fauna on the continental shelf [8]–[10]. *Chorismus antarcticus* Pfeffer, 1887 [11] (Hippolytidae) and *Notocrangon antarcticus* Pfeffer, 1887 [11] (Crangonidae) are the most abundant shelf inhabiting Antarctic shrimps [5], [10] and distributed around the Antarctic continent [9], [10], [12]. Abundance values confirm a preference for depths ≤400 m by *Chorismus antarcticus* (up to four specimens per m^2^) and 200–600 m by *Notocrangon antarcticus* (up to three specimens per m^2^) [10]. *Chorismus antarcticus* may occasionally be found in the Magellan region, but *Notocrangon antarcticus* has been recorded north of the Antarctic convergence only once [13]. While both of these species represent typical and abundant Antarctic shelf or slope species, the deep-sea shrimp *Nematocarcinus lanceopes* Bate, 1888 [14] is known from the deep sea around Antarctica to approximately 4,000 m water depth, sub-Antarctic islands as well as other adjacent deep-sea basins off Chile and South Africa [5], [14]–[20]. As a part of extensive studies of the benthic fauna of the Weddell Sea, up to nine specimens per m^2^ were recorded between 500 and 1200 m depth, revealing a broad bathymetric distribution and high densities of specimens on the Antarctic shelf [9], [10]. Nevertheless, beside fragmented information of their biogeographic distribution we have only poor knowledge of the biology of Antarctic Caridea. So far, most studies analysed aspects of reproductive biology and larval development [12], [21]–[26], biochemical or metabolic characteristics [27]–[34], the digestive system [35], as well as their infestation by ectoparasites [36]. A first pioneering phylogeographic study analysing various populations of *Chorismus antarcticus* and *Nematocarcinus lanceopes* gave evidence for a postglacial expansion of the shelf-inhabiting species *Chorismus antarcticus* [37], though a few potential refugial areas may have remained on the shelf [38], [39]. In contrast, populations of the deep-water shrimp *Nematocarcinus lanceopes* were less affected in their genetic diversity, supporting a scenario that recent and recurrent glaciations of the continental shelf are very likely to have affected benthic shallow-water shelf species generally far more than pelagic species or primarily deep-sea distributed species [40].

In order to understand the fragmented information of biogeography and spatial distribution of these three shrimp species, we developed Species Distribution Models (SDMs) based on a most comprehensive set of species records and current environmental conditions. SDMs are based on the theoretical concept that every species occupies a characteristic fundamental niche, wherein it's realized distribution is commonly restricted by biotic interactions and dispersal limitations [41]. Climatic conditions have a major impact on continental scales [42], as they affect not only the species directly but also its biotic environment [43] (see BIOCLIM); [44]. The coherency between observation of species ecological properities and their distribution is known in the terrestrial and aquatic environment [45], [46] and recent development of new algorithms enabled to assess the coherences between environmental conditions and species distribution patterns [42], [47]–[50].

During the last few years, SDMs have been successfully applied in the terrestrial environment [47], [48], [51] and recently also used in studying distribution of marine species [52]–[57]. Possible applications comprise e.g. studies of likely future climate change effects on global fish biodiversity [52], [53], distribution of whales in the mediterranean [58] and Antarctic waters [59] or assessment of possible glacial refugia and population fragmentation of the Atlantic cod [60].

Herein, we use SDMs to assess the potential distributions of three Antarctic shrimps for a current and a last glacial maximum (LGM) scenario around the Antarctic continent for the first time. This approach allows us to examine their current potential distribution patterns and gain information about possible glacial refugia during times with unfavorable conditions on the Antarctic shelf.

## Materials and Methods

### Species records and environmental data

Species data points were compiled through various sources, e.g. the Global Biodiversity Information Facility (GBIF, www.gbif.org), Ocean Biogeographic Information System (OBIS, www.iobis.org), SCAR-MarBIN (http://www.scarmarbin.be), and a comprehensive literature review as well as Antarctic Expedition cruise reports (Supp.Tab1.). All data were checked for redundancy or errors, e.g. erroneous GPS coordinates. Species records were located all around Antarctica with regard to different sampling effort of the expeditions in some regions. Therefore, our final data sets comprised of 93 records for *N. lanceopes*, 100 for *C. antarcticus* and 151 for *N. antarcticus*.

Marine Environmental data with a spatial resolution of 5 arcmin were obtained from Bio-ORACLE (www.oracle.ugent.be) and interpolated from AquaMaps (http://www.aquamaps.org/download/main.php). Ocean depth information was obtained from ETOPO1 (www.ngdc.noaa.gov) and re-sampled to the same resolution of 5 arcmin using ESRI ArcGIS 10.0 To develop paleo-climatic scenarios we obtained respective environmental information from Glacial Ocean Atlas [61], which was also re-sampled to the same resolution. Glacial ocean bottom temperature based on the findings of core analyses [62] (http://pmip2.lsce.ipsl.fr/).

We tested the inter-correlation structure among all predictor variables as high inter-correlations may negatively affect SDM performance and its transferability through space and time [63], [64]. Herein, we chose five environmental variables with R^2^<0.75 based on pair-wise correlation analyses using squared Pearson's correlation. Variables used in our models were sea ice coverage (icecov), depth (depth), annual mean sea surface temperature (SSTmean), annual mean salinity (salinity), and annual mean bottom temperature (sbt). All of them were suggested to be putatively suitable for large-scale species distribution models and hind casting projections [54], [60]. Environmental profiles were generated in R [65] with the sm.density.compare function from the sm package [66].

### Species distribution models

SDMs based on the species records and the five environmental variables were computed for the three species using Maxent version 3.3.3e applying the default settings [49], [67], [68]. Maxent per default requires random background data points, which are ideally situated in potentially colonizable areas for the target species [69]–[71]. In this context, the selection of appropriate background data represents an important step in model building and can affect the SDM performance [69], [70], [72]. Here, we included as background a smoothed buffer of 1000 km around species records plus adjacent areas, which are likely to be reached by ocean currents due to the fact that the exact range of all analyzed species is unknown. Although a restriction of the environmental space used for model training is pivotal for a good discrimination ability of the SDM, projections beyond the training range in space or time may be associated with an increased uncertainty. Therefore, we quantified the spatial distribution of non-analogous environmental conditions via multivariate environmental similarity surfaces (MESS, [73]). MESS maps were computed for current and paleo scenarios, which highlight those areas where at least one predictor exceeds the conditions available within the training range of the SDM.

For model testing, we randomly omitted 25% of the species records from model training and performed 100 Bootstrap replicates. As a test for predictive performance of the SDMs, Maxent automatically calculates two different versions of the so-called ‘Area Under the receiver operation characteristic Curve’ (AUC). Generally, AUC scores represent the ability of the model to distinguish presence data from background and range from 0.5 (random distribution, model without predictive ability) to 1.0 (model gives perfect predictions) [74], [75]. Test AUC scores quantify the model's ability to capture the randomly omitted records. In this study we used a logistic Maxent output format giving a continuous range from 0 (unsuitable environmental conditions) to 1 (optimal conditions) [68], and a minimum training presence logistic threshold as a non-fixed threshold as proposed by Liu et al. [76].

## Results

**Figure 1:**
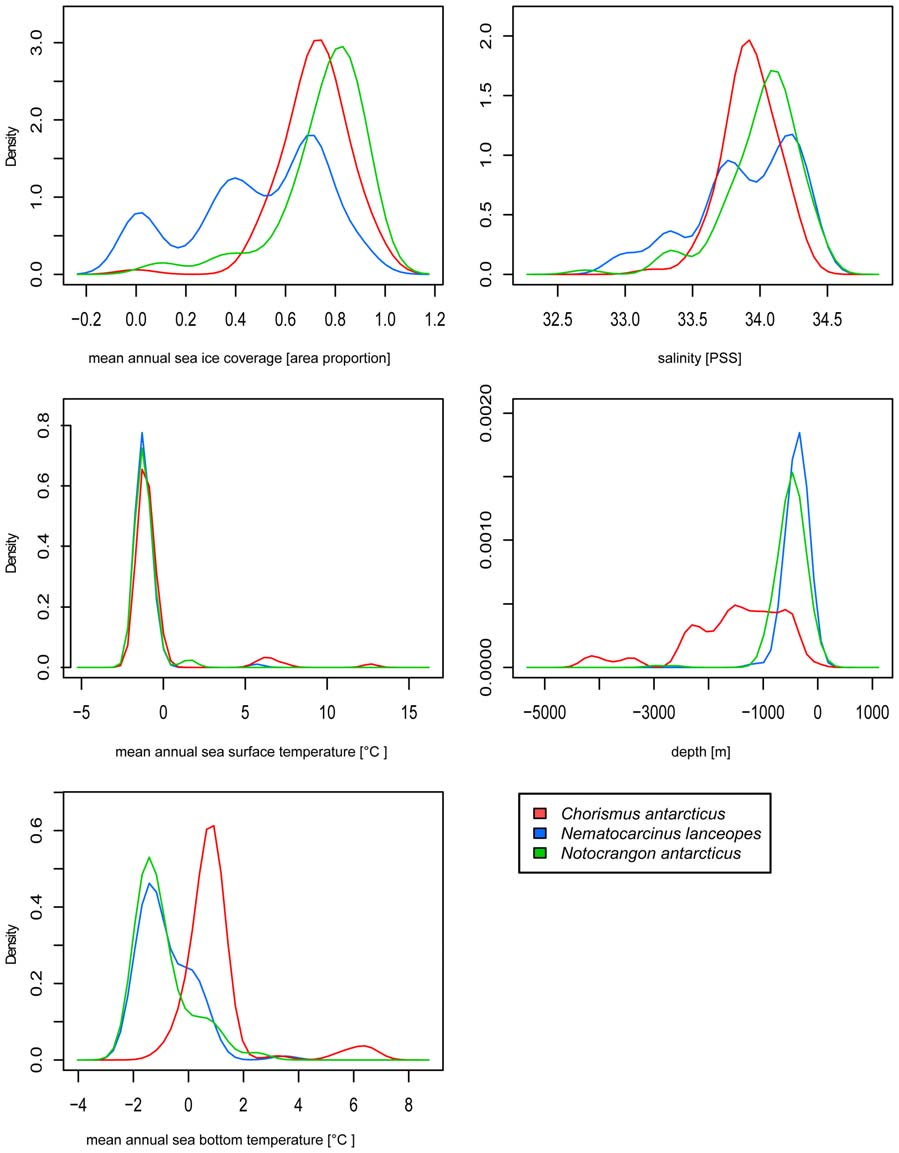
Environmental profiles. Environmental conditions at sample localities for *C. antarcticus*, *N. lanceopes* and *N. antarcticus*.

Environmental profiles in Fig. 1 illustrate the tolerances of the species in different environmental dimensions. Here, the most apparent differences between *N. lanceopes* and the other species are the lower tolerance for annual sea ice coverage and bottom temperature as well as a strong preference for deeper waters.

### Performance of SDMs and current potential distribution

**Figure 2:**
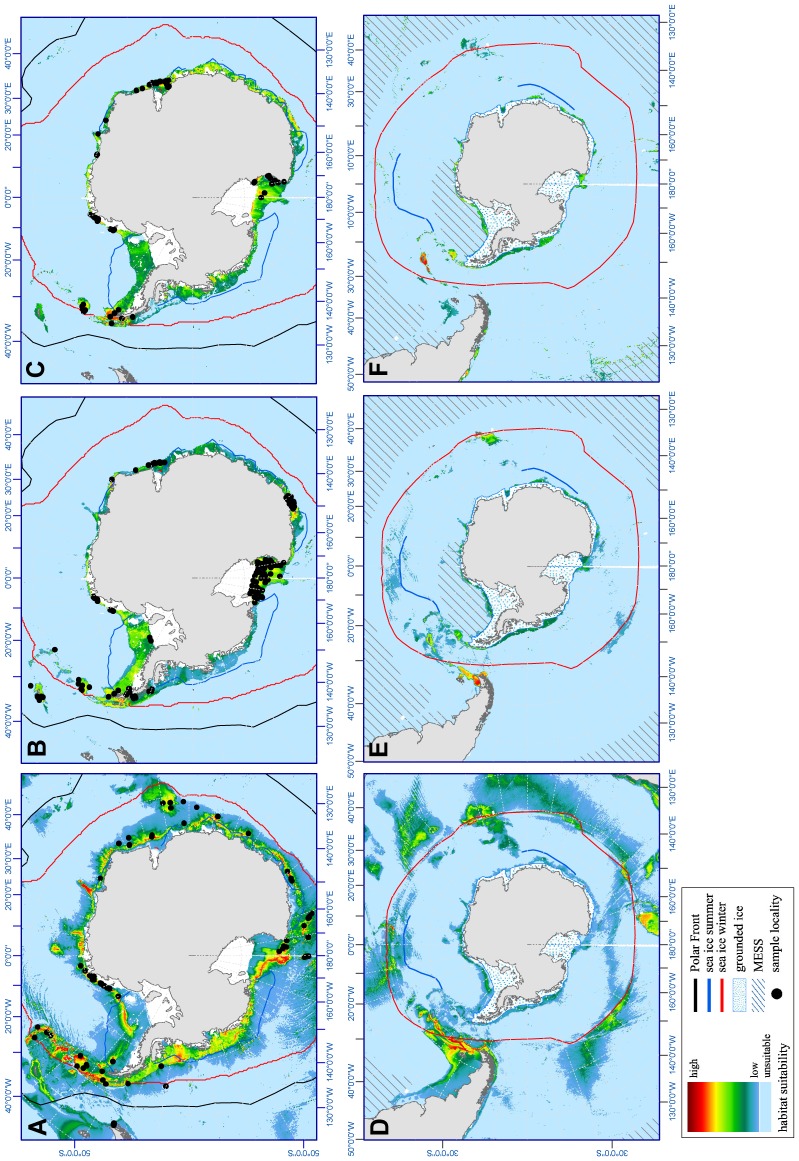
Present and paleo potential distribution maps. The potential distribution of the Antarctic decapod shrimps *N. lanceopes*, *N. antarcticus* and *C. antarcticus* computed with Maxent 3.3.3e derived from current conditions (A–C) and projected onto a Last Glacial Maximum scenario (D–F). Habitat suitability ranges from low (blue) to high (red). Also shown are the summer and winter sea-ice extent and the Polar Front. Shaded areas (MESS) indicate climate conditions out of the species range.

Our SDMs received excellent AUC values for all three species. Mean test AUC for 100 computed SDMs was 0.96 for *Nematocarcinus lanceopes*. For this species, ‘depth’ had the highest explanative power (42.8%), followed by ‘icecov’ (42.1%), ‘sbt’ (8.0%) and ‘SSTmean’ (6.1%), while salinity had a relatively low contribution value (1.0%). Average minimum training presence is 0.02 and 10 percentile training presence is 0.13. According to our SDM, the current potential distribution of *Nematocarcinus lanceopes* comprises the shelf areas and slopes of Antarctica with the Antarctic Peninsula, South Georgia ridge, South Orkney and South Sandwich Islands, the Kerguelen Plateau, the Pacific-Antarctic Ridge, the western Ross Sea near Balleny islands as well as parts of the Chilean west coast (see Fig. 2 A).

The SDMs computed for *Chorismus antarcticus* had a mean test AUC of 0.98. Here, average ‘icecov’ had the highest explanative power (51.4%), followed by ‘depth’ (42.4%), ‘sbt’ (3.7%), ‘salinity’ (1.4%) and ‘SSTmean’ (0.9%). Thresholds (minimum training and 10 percentile training) were 0.10 and 0.29. The current potential distribution of *Chorismus antarcticus* comprises the lower shelf areas of Antarctica, the Scotia Arc and South Georgia, the shelf areas of sub-Antarctic islands, Ross Sea shelf and lower parts of the Kerguelen Plateau (see Fig. 2 C).

Finally, SDMs computed for *Notocrangon antarcticus* had a test AUC of 0.98. The variable with highest explanative power was ‘depth’ (66.6%), followed by ‘icecov’ (21.8%), ‘salinity’ (7.6%), ‘sbt’ (2.5%) and ‘SSTmean’ (1.8%). Thresholds (minimum training and 10 percentile training) were 0.04 and 0.38. The SDM for *Notocrangon antarcticus* showed a potential distribution similar to *Chorismus antarcticus* but with a little shift to the deeper shelf areas (see Fig. 2 B).

### Projections for a Last Glacial Maximum scenario

Our SDM projections for the Last Glacial scenario (21 ky BP) suggest a partial shift of the potential distributions to lower latitudes for all three analyzed species. In Fig. 2 D–F, unsuitable shelf areas covered by grounded ice [77] are blue shaded.

The LGM projection for *Chorismus antarcticus* indicate suitable areas in those parts of the Antarctic shelf which were probably not completely covered by ground ice (Anderson et al. 2002). Further areas with high suitability were located around South Georgia and the sub-Antarctic islands as well as small patches on the tip of South America (Fig. 2 F). The projection of the potential distribution of *Notocrangon antarcticus* suggested suitable areas around South Georgia, the South Sandwich Islands, Falkland Islands and the southern tip of South America as well as parts of the Kerguelen Islands (Fig. 2 E). In contrast to both shallow-water species, our projection for the deep-sea shrimp *Nematocarcinus lanceopes* gave evidence for a lower suitability on the Antarctic shelf but also revealed areas with higher suitability on a circle alongside the area of the LGM ice extent, connecting the sub-Antarctic islands as well as ocean ridges and plateaus between the 59th and 45th latitude. Here, areas downward to depths of 4000 meters around South Georgia and Bouvet Island, northern parts of the Kerguelen Plateau, the Tasmania and Campbell Plateau were indicated as environmentally suitable areas during the LGM (Fig. 2 D). For times of the LGM the Weddell Sea exhibits non-analogous environmental conditions exceeding those of the present training range of *C. antarcticus* and *N. antarcticus*. Here, salinity was identified as the most dissimilar variable.

**Figure 3:**
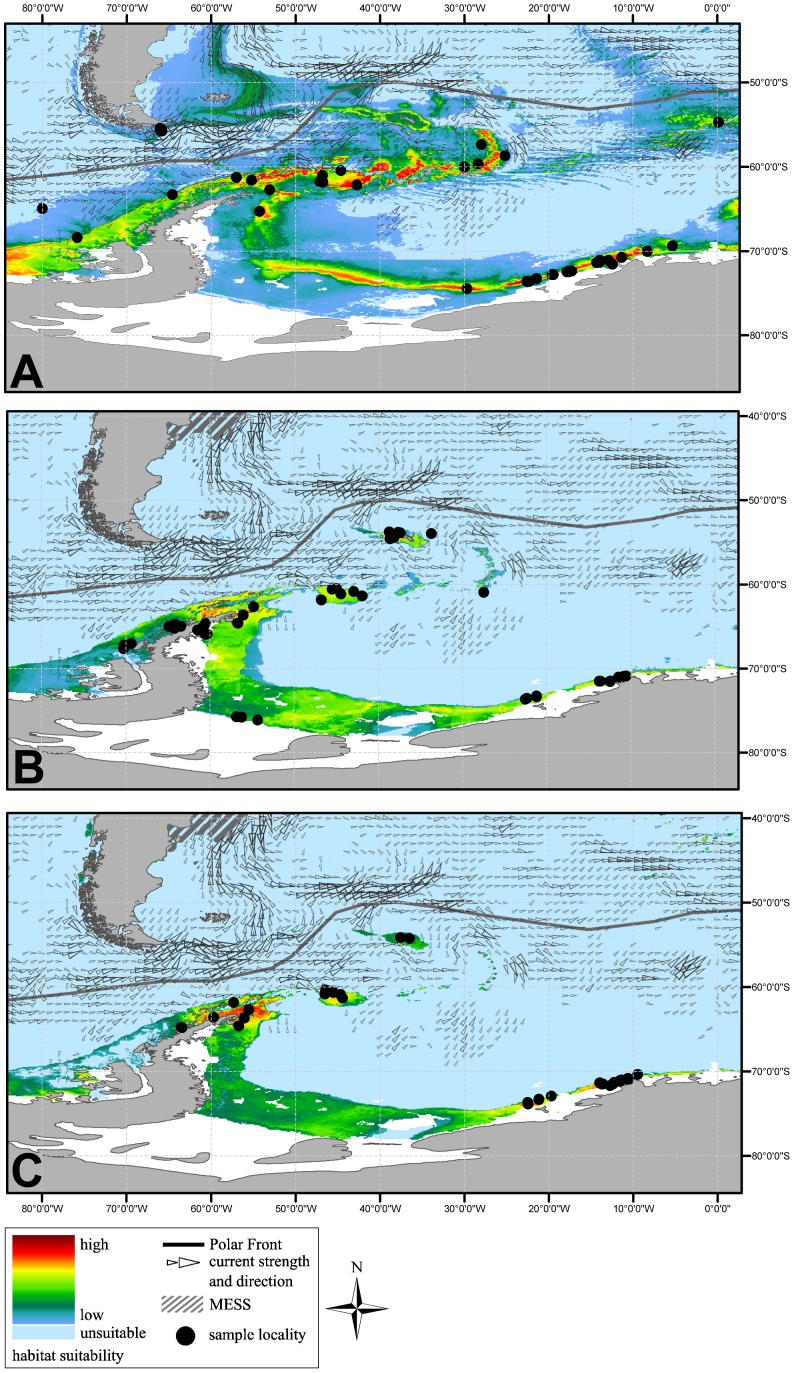
Present potential distribution maps. The potential distribution of *N. lanceopes*, *N. antarcticus* and *C. antarcticus* (A–C) computed with Maxent 3.3.3e derived from current conditions. Display window for the area Weddell Sea and Antarctic Peninsula. Indicated the early summer near-surface currents [78], [79], which are likely to affect the drift of larval stages. Shaded areas (MESS) indicate climate conditions out of the species range.

A closer look on the current habitat suitability in the Weddell Sea and Antarctic Peninsula between 84° west and 3° east is provided in Fig. 3. Here, the early summer near-surface currents were indicated to assess the direction and accessibility of larval drifted distribution by currents when spawned in these areas [78], [79]. Currently known occurrences and suggested habitats for *N. antarcticus* and *C. antarcticus* were located south off the Polar Front (except samples of *C. antarcticus* from Prince Edward Island). For *N. lanceopes*, model suggestion and sample localities were also found north of the Polar front from “Tierra del Fuego” and the western Chilean coast. Nevertheless, the habitat suitability is much lower here.

## Discussion

This study is the first approach to model the biogeographic distribution patterns of benthic shallow-water and deep-sea arthropods in the Southern Ocean covering their current distribution and a hind casting projection. Although first molecular studies already provided clear evidence of homogenous genetic identity in circum-Antarctic distribution for both *N. lanceopes* and *C. antarcticus* [37], a detailed assessment of their distribution patterns was not given. Our SDMs complete the so far only fragmented information about the potential distributions of *N. antarcticus*, *C. antarcticus and N. lanceopes* around the Antarctic continent. Modeling projections for the LGM give evidence for a population reduction affecting genetic diversity in shallow water shrimp species (c.f. [37]), and a northward shift but less restricted range for the deep-sea shrimp species.

### Models and data

Species records of all three shrimp species were included, comprising various regions on the Antarctic shelf, sub-Antarctic islands and also on southern parts of South America. Our models are based on an adequate number of species records and display the complete width of the environmental range of the species across the currently realized distribution on a broad scale. However, some areas in the Antarctic Ocean, the Amundsen Sea or eastern Ross Sea are overall not well explored in terms for decapods and benthic communities. Therefore, the information on suitable habitat for Antarctic shrimps provided here may serve as a useful baseline for future studies of those regions.

The choice of reasonable parameters for a SDM approach is crucial and depends on the general question of the study, the examined taxa and the availability of parameters for different projections in time and their spatial extent [47], [48], [54], [63], [80], [81]. In our study we used a set of parameters that were suggested to be suitable for large-scale geographic models and available for a current and a paleoclimatic scenario. In this context, bathymetry plays an important role in directly or indirectly affecting the environmental conditions for marine organisms, such as pressure, availability of primary production, temperature, and others [82], [83]. Beside bathymetry, sea ice coverage and sea surface temperature are an important predictor and influence the primary production and therewith the food availability for all pelagic and benthic communities in the deeper water zones [55], [84], [85]. For *N. lanceopes*, the mean annual sea-ice coverage is the most important predictor. Salinity demonstrated a relatively low explanative power as a predictive variable. However, for *N. antarcticus* it seemed to have a higher contribution (7.6%) than for *N. lanceopes* or *C. antarcticus* (1–1.4%). *N. antarcticus* had a higher tolerance for salinity (see Fig. 1).

Identified areas of non-analogous environmental conditions for *C. antarcticus* and *N. antarcticus* are likely to base on the higher salinity in LGM environmental data for the Weddell Sea. However, this topic is still under debate and there are different scenarios and anomaly models for the LGM salinity of the Weddell Sea [61].

For *N. lanceopes* our modeling suggests highly suitable areas on the Antarctic slopes and around the sub-Antarctic islands. Although areas with highest suitability were suggested between 1500 and 3000 m, areas with a lower suitability score were found downward to 4500 meters. We found similar patterns for *C. antarcticus* and *N. antarcticus*, with various suitable areas on the Antarctic shelf connected by small corridors and around the sub-Antarctic islands. This pattern of closely linked suitable areas is concordant with the comprehensive molecular data that revealed genetic homogeneity based on mtDNA and no evidence for a geographical substructure around Antarctica and the sub-Antarctic islands for *N. lanceopes* as well as *C. antarcticus* [37].

### Potential refuges during the Last Glacial Maximum

Sea ice is an important factor affecting the distribution of numerous marine species in Antarctica. Extensive sea ice coverage reduces the photosynthetically driven primary production [84] and therewith the survival probability of planktotrophic larvae, although sea ice coverage does not necessarily preclude all life under the ice. For example, larvae of the Antarctic krill (*Euphausia superba*) for example are known to feed on sea ice algae under and on the edge of sea ice [86] and ice drilling on the Shelf 100 km from the coastline revealed a so far unexpected benthic suspension feeder community [87]; for discussion see also [88].

However, in times characterized by extreme climatic conditions like the LGM, a thick multiannual sea ice layer and additional snow cover throughout the year was likely to restrict benthic life in higher latitudes or at least force it to retreat to a few areas with favorable conditions like we know from present day coastal or open-ocean polynyas [39], [44], [84]. It has also been suggested that some possible open ocean polynyas could have nourished marine organisms in regions with multiannual sea ice coverage, acting as “glacial refugia” for shelf-inhabiting communities [39]. Regarding the potential distribution of *C. antarcticus* in the LGM, our models suggested the presence of refugial areas around the southern tip of South America, South Georgia and the Kerguelen plateau. It should be noted however, that both *C. antarcticus* as well as *N. lanceopes* may have faced ecological competition with congeners e.g. in the South Atlantic, *C. tuberculatus* and *N. longirostris*, respectively. Areas on the Antarctic shelf, which are suggested to be suitable for *C. antarcticus* during the LGM, should be regarded with caution because effects of scouring icebergs or lack of food due to extreme distances to the sea ice front were not considered in the models yet. Furthermore, large parts of the shelf habitats that are currently inhabited by *C. antarcticus* and *N. antarcticus* were occupied by grounding ice masses at the LGM [38], [77], [89]. Evidence for a survival of species on the shelf during the LGM has also been suggested by molecular genetic data on benthic direct-developing invertebrates [90], [91]. While the pelagic larvae of decapods have a higher motility than the offspring from brooding species and can be easily distributed by ocean currents, a scenario of a relatively fast re-colonization of ice freed shelf areas during interglacial periods from a few refugial areas seems more plausible [39]. Evidence from molecular data also indicates a late Pleistocene bottleneck and a recent population expansion for *C. antarcticus* [37].

In contrast to the more restricted habitat of the two shelf species the predicted LGM habitat of *N. lanceopes* reaches down to the abyssal plains of the Southern Ocean on a circle alongside the ice margins. Though low in suitability, this habitat distribution pattern along the ice margin may have allowed feeding and successful development of pelagic larvae during the LGM [12]. Here, a higher primary productivity and upwelling processes could have provided nutrient-rich waters, supporting feeding and reproduction, and advection processes may have supported biological activity in parts of the adjacent multi-annual sea-ice zone (for discussion see [39], [88]. Furthermore, these advection processes may have reached beyond the ice margins and may have enabled suitable conditions. However, the precise LGM sea-ice extent is unknown and subject to discussion, and various scenarios based on different core analyses do exist. In this context, various data indicate a lower LGM summer sea-ice extent around eastern Antarctica. [92]. If the aforementioned areas around the Antarctic Peninsula and sub-Antarctic islands were the main refugial areas for *C. antarcticus* and *N. antarcticus*, we would expect a higher genetic diversity in these areas (e.g. in terms of haplotype diversity) compared to the populations found on the shelf [93]. Contrarily, a specific pattern of genetically more diverse refugial areas may be blurred and mixed up again by gene flow when larvae distribution is fast and extensive. However, the genetic pattern of populations from suggested refugial areas around South American could not be tested in the present study due to the lack of suitable preserved specimens for molecular studies [37].

### Population connectivity

Ocean currents play an important role for transporting larvae from source areas to others and therefore can support a constant dispersal of larvae even between distant populations [94]. Few studies showed attempts to calculate larval dispersal of pelagic fish and invertebrate species [53], [95], [96]. Dispersal models typically assume a passive dispersal and diffusion and incorporate the strength and direction of ocean currents as well as pelagic larval duration. Although a few studies gained insight in Antarctic decapod larval biology [7], [12], a detailed knowledge of spawned numbers and distribution areas is still unknown.

In the case of Antarctic krill (*Euphausia superba*), a recent study revealed a homogeneous genetic pattern and suggests an active role of the Antarctic Circumpolar Current (ACC) to disperse and mix up populations around the Antarctic continent [97]. Shared mitochondrial haplotypes for *N. lanceopes* and *C. antarcticus* in locations on the Antarctic shelf and several sub-Antarctic islands also support a scenario of population connectivity and panmixia driven by ocean currents [37]. Larvae of all three species are planktotrophic and require food availability over several months for a successfully complete development [12], [38].

For *N. lanceopes*, there is evidence for a larval development connected to opening of early summer polynyas where sufficient food resources are available [12]. Adult females carry relatively big eggs and larvae are large and advanced at hatching. One suggestion of a possible transport from larvae hatching in deep waters to the shallower euphotic zone is by upwelling currents [38]. Once in the upper water levels, larvae are likely to be transported with the predominant currents.

Our models suggest connected patches of highly suitable areas for *N. lanceopes* (Fig. 3 A) ranging from the tip of the Antarctic Peninsula and South Shetland Island via the South Orkney Islands up to the Scotia Arc. Here, predominantly the near surface currents run along these habitat patches in eastern direction and are likely to support a transport of larvae from western to eastern populations.

Genetic evidence for long distance dispersal and a “Sub-Antarctic islands hopping” from west to eastern direction was also found for the isopod *Septemserolis septemcarinata* [98], indicating the importance of the ACC even for organisms with no pelagic stages. On the other hand, strong currents such as the ACC in the Drake Passage can function as an effective boundary between populations or species and connectivity especially for benthic organisms without pelagic larvae can be even more reduced when no suitable corridors are available, e.g. temperature or depth is unsuitable [99]. Species with genetically distinct clades between South America and Antarctica for example were found for ophiuroids [100], ribbon worms [101] and bivalves [102]. In the case of *N. lanceopes* and *C. antarcticus* molecular data from South America are currently missing, but strong ocean currents through the Drake Passage at times when larvae are spawned may act as a barrier and restrict direct gene flow between Antarctic and sub-Antarctic populations compare to those in and South America on the other side of the ACC. The existence of congeners of both species in the South Atlantic (*C. tuberculatus*, *N. longirostris*) [103], however, may be also a strong hint of restricted gene flow across the ACC.

## Supporting Information

List S1 **Antarctic Expeditions and cruise reports.**
(DOCX)
