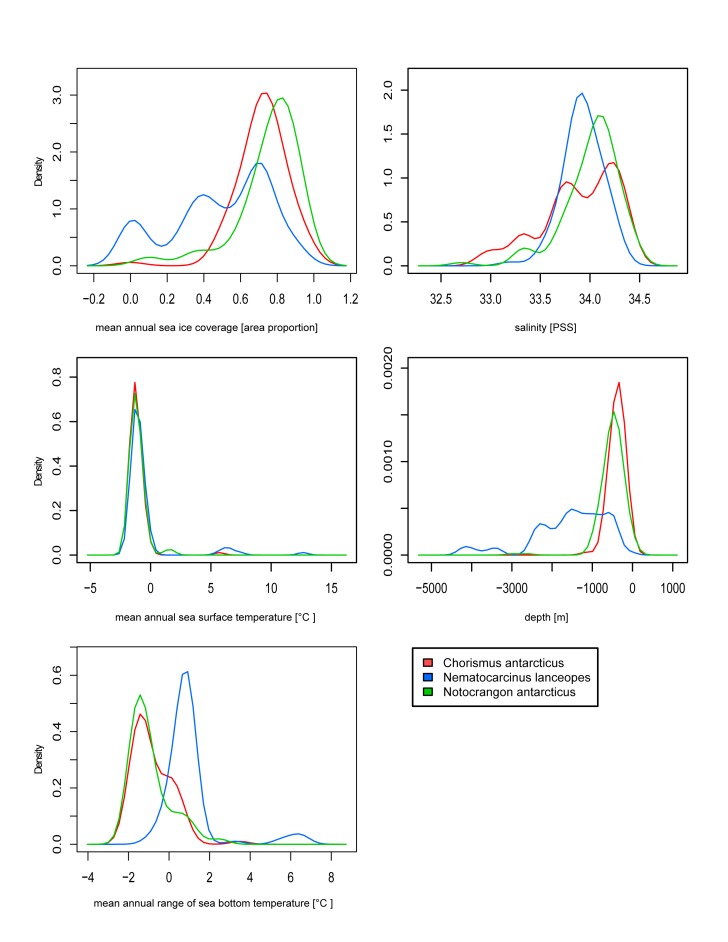# Correction: Effects of Late-Cenozoic Glaciation on Habitat Availability in Antarctic Benthic Shrimps (Crustacea: Decapoda: Caridea)

**DOI:** 10.1371/annotation/0c5390b8-72b0-4b7e-85a3-b8c0fd9f62bf

**Published:** 2013-03-04

**Authors:** Johannes Dambach, Sven Thatje, Dennis Rödder, Zeenatul Basher, Michael J. Raupach

There is an error in Figure 1. The correct Figure 1 can be seen here: 

**Figure:**