# Supplementary material for: Effects of Late-Cenozoic Glaciation on Habitat Availability in Antarctic Benthic Shrimps (Crustacea: Decapoda: Caridea)
Source: PLoS One. 2012 Sep 27;7(9):e46283. doi: 10.1371/journal.pone.0046283 (PMC3459913; doi:10.1371/journal.pone.0046283)
Supplement: List S1 — Antarctic Expeditions and cruise reports. (DOCX) [file pone.0046283.s001.docx]

**Effects of Late-Cenozoic glaciation on habitat availability in Antarctic benthic decapod crustaceans**

Johannes Dambach^1*^, Sven Thatje^2^, Dennis Rödder^1^, Zeenatul Basher^3^, Michael J. Raupach^4^

**1** Zoologisches Forschungsmuseum Alexander Koenig, Bonn, Germany **2** Ocean and Earth Science, National Oceanography Centre, University of Southampton, Southampton, United Kingdom **3** Leigh Marine Laboratory, University of Auckland, Auckland, New Zealand, **4** Deutsches Zentrum für Marine Biodiversitätsforschung, Senckenberg am Meer, Wilhelmshaven, Germany

* E-mail: j.dambach.zfmk@uni-bonn.de

**Supplement list 1: Antarctic Expeditions and Cruise Reports**

Arntz WE, Brey T (2003) The expedtion ANTARKTIS XIX/5 (LAMPOS) of RV "Polarstern" in 2002. Rep Polar Mar Res 462:1-120.

Arntz WE, Brey T (2005) The expedtion ANTARKTIS XXI/2 (BENDEX) of RV "Polarstern" in 2003/2004. Rep Polar Mar Res 503:1-149.

Fahrbach E (2006) The expedition ANTARKTIS-XXII/3 of research vessel “Polarstern” in 2005.Rep Polar Mar Res 533:1-246.

Fütterer DK, Brandt A, Poore GCB (2003) The expeditions ANTARKTIS-XIX/3-4 of the Research Vessel POLARSTERN in 2002. Rep Polar Mar Res 470:1-174.

Gutt J (2008) The Expedition ANTARKTIS-XXIII/8 of the Research Vessel "Polarstern" in 2006/2007. Rep Polar Mar Res 569:1-152.

Kattner G (1998) The expedition ANTARKTIS XIV/2 of RV "Polarstern" in 1996/97. Rep Polar Res 274:1-87.

Mitchell J, Clark M (2004) Voyage report TAN04-02, Western Ross Sea voyage 2004. NIWA, Wellington.

RV Tangaroa, New Zealand IPY-CAML Voyage (Cruise TAN0802, 12Feb-11Mar 2008).
